# Spontaneous Haemothorax as Initial Presentation of Pleural Ewing Sarcoma: A Case Report

**DOI:** 10.1155/crpu/2865406

**Published:** 2026-05-20

**Authors:** Alishba Khan, Andrew Mcdougall, Jon Tomas, Saddam Ibrahim, David Snead, Joshua Dale, Beatriz Lara

**Affiliations:** ^1^ Department of Respiratory Medicine, University Hospital Coventry and Warwickshire NHS Foundation Trust, Coventry, UK; ^2^ Department of Palliative Care Medicine, University Hospital Coventry and Warwickshire NHS Foundation Trust, Coventry, UK; ^3^ Department of Pathology, University Hospital Coventry and Warwickshire NHS Foundation Trust, Coventry, UK

**Keywords:** Ewing sarcoma, haemothorax, metastatic cancer, pleural malignancy

## Abstract

This case report discusses a middle‐aged patient with metastatic pleuro‐pulmonary Ewing sarcoma, with the rare initial presentation of spontaneous haemothorax. The patient had a prior history of Ewing sarcoma of the pelvis in young age, which had been treated with chemoradiotherapy with remission. There was a 2‐week history of right sided chest pain and shortness of breath, and a chest x‐ray showed complete opacification of the right hemithorax, with subsequent CT arising suspicion of a heterogeneous soft tissue pleural lesion with fluid in the pleural cavity. While awaiting further treatment including biopsy, the patient deteriorated rapidly and was eventually started on end‐of‐life care. Postmortem examination showed large right pleural haemorrhage with lung collapse, and histological examination of pleural lesions confirmed a diagnosis of recurrent Ewing′s sarcoma. Extraskeletal presentations of Ewing′s sarcoma, particularly with spontaneous haemothorax as the initial presentation have been rarely reported in the literature in adult patients. Diagnosing pleural Ewing′s sarcoma requires a multimodal approach, utilising investigations such as contrast‐enhanced CT imaging, pleural fluid analysis, thoracoscopy, and PET‐CT to evaluate for disease spread. Treating pleural Ewing′s sarcoma can be challenging; early diagnosis and management of complications such as haemothorax is essential with thoracocentesis or thoracotomy. Ongoing treatment can include multimodal therapies including chemotherapy and radiotherapy with or without surgery, depending on disease extent.

## 1. Introduction

Pleural malignancies can have variable and often nonspecific clinical presentation, with symptoms such as cough, chest or back pain and exertional dyspnoea [1]. However, rarely, they may also present with spontaneous haemothorax, defined as haemothorax occurring in the absence of any traumatic force. This may be seen as a part of hemopneumothorax, vascular malformations, clotting disorders and/or neoplasia [2]. Although it is not uncommon to see haemorrhagic pleural effusions with various malignancies, the term haemothorax is used only when pleural fluid haematocrit is more than 50% of the peripheral blood haematocrit.

Pleural sarcomas are rare pleural malignancies [3], and reports in literature of a pleuro‐pulmonary presentation of Ewing sarcoma are even more so [4]. Here we present a case report of metastatic Ewing sarcoma, with pleuro‐pulmonary involvement, showcasing as spontaneous haemothorax in a middle‐aged patient.

## 2. Case Report

A middle‐aged (58‐year‐old) Caucasian male presented to the emergency department with a 2‐week history of right‐sided lower chest pain, cough and shortness of breath. The patient had previously been treated for right‐sided pelvic Ewing sarcoma in 1989 with chemotherapy and radiotherapy at University Hospital Birmingham and had remained in remission for this. Their past medical history was also significant for hypertension, Type 2 diabetes mellitus and chronic kidney disease Stage 3. They had been in good physical health prior to the current presentation, with an Eastern Cooperative Oncology Group (ECOG) performance status score of 0. They had known allergies to vancomycin, erythromycin and clarithromycin.

On initial presentation they were noted to be tachycardic with a pulse rate of 136/min but were otherwise hemodynamically stable with normal blood pressure and normal oxygen saturation on room air. They were alert and oriented, and physical examination only showed decreased air entry on the right side of the chest, with an unremarkable cardiac and abdominal examination. Bedside investigations showed sinus tachycardia on his ECG. They were noted to have significant metabolic acidosis on venous blood gas, with a pH of 7.283, lactate 2.3, bicarbonate 19.3 and base excess of –4.1.

**Figure 1:**
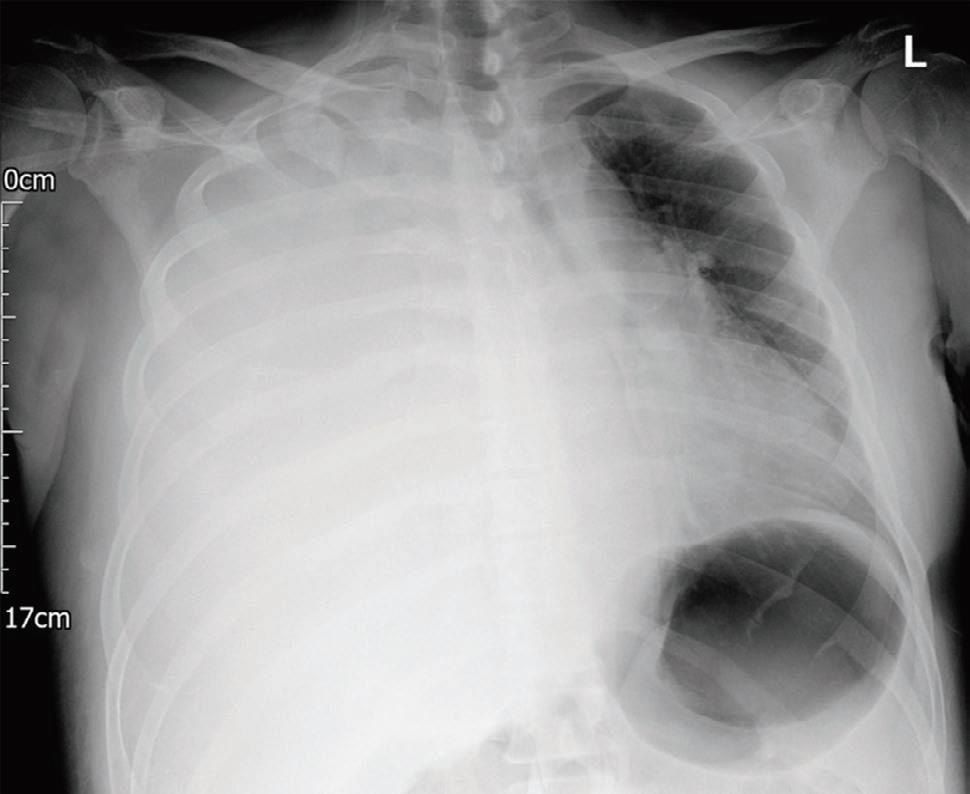
Chest x‐ray with complete opacification of the right hemithorax and tracheal deviation to the left side.

Initial blood investigations showed neutrophilia (white cell count 15.8 × 10^9^; neutrophil count 13.0 × 10^9^), with raised C‐reactive protein (366 mg/L); his haemoglobin and platelet count were within normal limits. They were also noted to have a deranged clotting profile with an international normalized ratio (INR) of 1.7, with slightly raised D‐dimer levels (1.68 mg/L). Their serum creatinine (178 *μ*mol/L) and total bilirubin (26 *μ*mol/L) were also elevated. Viral respiratory screen as well as COVID‐19 screening was negative. They underwent chest x‐ray, which showed complete opacification of the right hemithorax (Figure 1). Bedside chest ultrasound reported a hyperechoic field with pockets of hypoechoic areas on the right side, with complete right‐sided effusion. However, there was uncertainty about the presence of underlying lung collapse or tumour.

**Figure 2:**
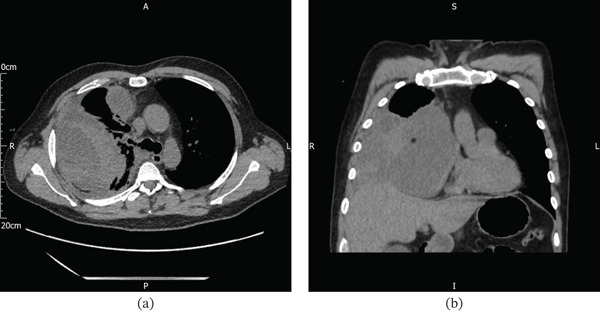
(a and b) Thoracic CT showing hyperdense pleural‐based lesions, with fluid and heterogeneous soft tissue.

The patient was admitted for further treatment and was started on intravenous co‐amoxiclav for presumed pneumonia. Due to previous suspicion of contrast allergy, they underwent CT thorax, abdomen and pelvis without contrast, which was compared with a previous CT from 2 years ago. This revealed multiple hyperdense pleural‐based lesions, with fluid and heterogeneous soft tissue and underlying right lung collapse (Figure 2). It also showed an enlarged lymph node in the fat posterior to lower sternum, measuring 12 mm. The abdominal organs were unremarkable, apart from an atrophic right kidney with gross hydronephrosis, which had been reported previously. CT also showed relatively stable appearances of the previously seen permeative changes in right hemipelvis, consistent with known Ewing′s sarcoma. Due to suspicion of pulmonary embolism in the noncontrast scan, they were scheduled for a PET CT scan and ventilation‐perfusion scan.

The patient was referred to the Royal Orthopaedic Sarcoma multidisciplinary team (MDT) meeting to discuss whether these symptoms could be due to metastasis of his Ewing sarcoma, and after discussion in the MDT they were advised to undergo a CT‐guided lung biopsy. However, the patient started to deteriorate rapidly; despite improvement in inflammatory markers, there was a drop in haemoglobin to 96 g/dL, and they developed acute kidney injury with creatinine rising to 274 *μ*mol/L. They also developed severe pain crisis and could not tolerate either the PET Scan or the VQ Scan. After palliative team review, a syringe driver was started with alfentanil, midazolam and haloperidol. Their deterioration progressed rapidly, and they became severely short of breath with oxygen saturations dropping to 76% on room air. They were treated with high‐flow oxygen. Blood gas showed significant metabolic acidosis with pH of 7.20, bicarbonate 15.1 and lactate 3.6. Repeat blood tests showed a major drop in their haemoglobin to 65 g/dL, and they were also noted to have reactive thrombocythemia (platelet count 559 × 10^9^), along with a sharp rise in inflammatory markers (white cell count 22 × 10^9^; CRP 369). Their clotting and renal profile were also severely deranged, with INR of 12 and creatinine of 330 *μ*mol/L. Despite transfusing 2 units of whole blood as well as injecting vitamin K, they continued to clinically deteriorate. Given the impending condition, they were decided to be for comfort care and not for resuscitation, and they later passed away on the same day.

**Figure 3:**
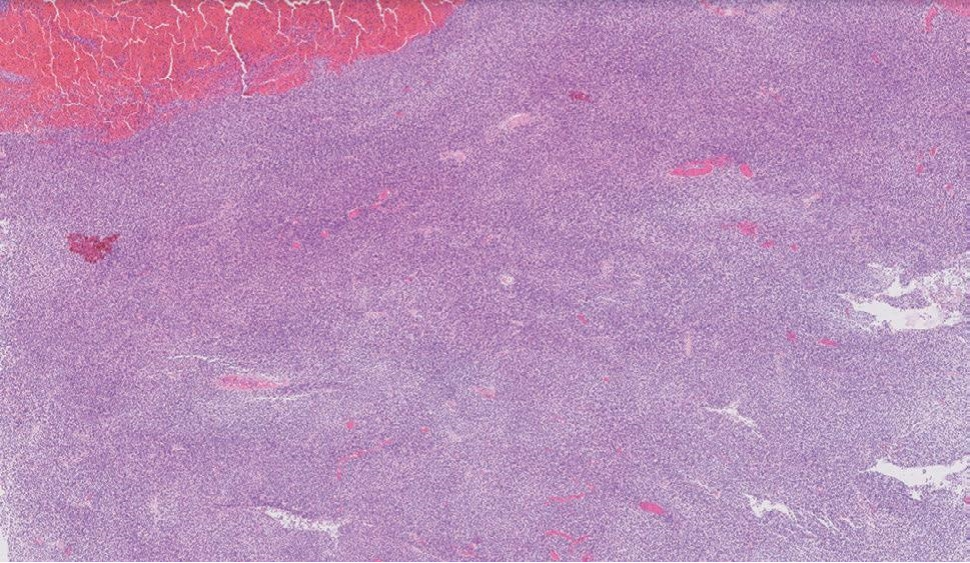
Histological appearance of the lesion at 2× magnification, showing a dense solid sheet of small round blue cells.

**Figure 4:**
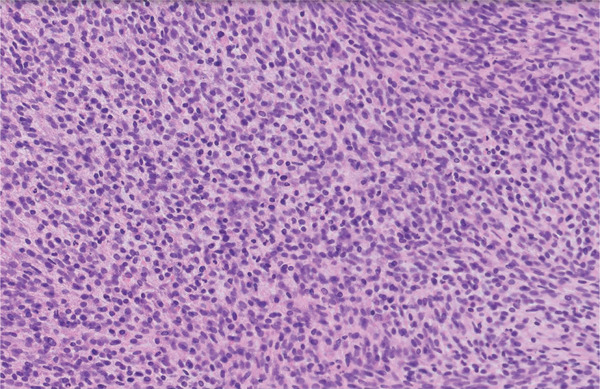
20× magnification. Microscopy shows a very cellular tumour consisting of sheets of uniform and hyperchromatic small round blue cells, some with a spindle cell morphology. The cytoplasm is scanty and pale‐eosinophilic, with indistinct cell borders. The tumour shows a high mitotic rate and many apoptotic bodies. The stroma is myxoid in places and necrosis is seen.

A limited postmortem examination of the neck and chest cavity was performed around 2 weeks after their death. This showed no external injuries, and normal pericardial cavity, heart, and the associated vasculature. It was noted that the right pleural cavity was filled with clotted blood weighing 1890 g, which had resulted in a partial collapse of the right lung, midline shift of the heart to the left, and partial collapse of the left lung. The haemorrhage appeared adherent to the pleural surface of the right middle lobe with several small foci of erosion of the pleural surface. No residual tumour was found at this point. Samples were then sent for histological analysis to determine the definite cause of the tumour: This showed a very cellular and extensive spindle celled tumour with areas of necrosis present throughout the material removed from the right pleural cavity (Figures 3 and 4). The tumour was found to not only be covering the pleural surface of the lung but also infiltrating the lung parenchyma and interlobular septae in multiple places. The morphology of the tumour was in keeping with that of Ewing′s sarcoma. Based on these findings and the clinical history of Ewing′s sarcoma in childhood, a postmortem diagnosis was made of recurrent Ewing′s sarcoma affecting the right pleural cavity and right lung with extensive haemothorax.

## 3. Discussion

Ewing sarcoma is a common childhood tumour, occurring commonly in the bones; however, it can also rarely present extraskeletally. The rarity of extra skeletal presentation of Ewing′s sarcoma can be seen by an incidence rate of 1 per 5–10 million [5]. Diagnosis of Ewing sarcoma of bone is best done by tissue biopsy to allow for histological analysis and detecting genomic translocation, supplemented by imaging. The presence of CD99+ cells on immunohistochemical staining and EWSR1 gene on chromosomal analysis is characteristic of this tumour [6]. Various imaging modalities can be used, such as MRI with gadolinium to outline the local involvement of the tumour; CT Chest to look for pulmonary metastasis; or PET CT for whole body scan of metastasis [7].

Treatment of Ewing sarcoma generally demands a multimodal approach, with a combination of systemic chemotherapy and local control with surgery versus radiotherapy [8]. Where complete en bloc removal of the tumour may not be possible, the choice tends to tilt in the favour of radiotherapy for local control of the tumour [9].

Only a few cases in literature report spontaneous haemothorax with Ewing sarcoma, with the majority reported in the paediatric population. Al‐Sukhni et al. reported a teenager presenting with acute chest pain and dyspnoea, who was noted to have a large multiloculated right‐sided pleural effusion on CT—insertion of a chest drain revealed massive spontaneous haemothorax, and further investigations after resolution of the haemothorax revealed malignant Ewing sarcoma [10]. In another case of a 12‐year‐old girl, spontaneous occurrence of haemothorax was the first presentation of insidiously growing extraskeletal Ewing sarcoma of the diaphragm [11].

Nontraumatic haemothorax as a presentation of metastatic disease in middle‐aged patients was reported in a case series as subsequent to malignant angiosarcoma of the bladder invading the subpleural space [12]; we did not find reported cases of spontaneous haemothorax secondary to Ewing sarcoma in this population.

Treatment of spontaneous haemothorax requires urgent intervention, with a focus on finding the source of bleeding to control it. Although thoracocentesis under cover of blood transfusion has been reported as the initial management of suspected haemothorax [10, 11], thoracotomy may be required for both diagnostic and therapeutic purposes. Ling et al. reported the case of a 15‐year‐old with a massive right‐sided pleural effusion with underlying lung collapse; thoracocentesis demonstrated massive haemothorax. An anterolateral thoracotomy was performed, which confirmed a haemorrhagic mass with pleuro‐pulmonary involvement, and histological analysis confirmed Ewing sarcoma. Whole body scan was negative for any other tumour deposits, and the patient underwent a successful course of chemotherapy [13]. This case demonstrates the importance of early surgical intervention to determine the cause of bleeding, and to aid further management with adjunctive treatment.

There were several limitations in our case. Initial treatment of the right pleural effusion reported on imaging was delayed due to a deranged coagulation profile despite the patient not being on any anticoagulant medications. Although the patient was planned for diagnostic investigations including PET CT, ventilation‐perfusion scan and imaging guided biopsy, these investigations could not be performed as they developed severe pain crisis despite intervening with continuous pain killers through a syringe driver. Due to rapid deterioration and development of acute kidney injury, sepsis and coagulopathy, the patient was not considered amenable for thoracocentesis or surgical exploration and they were treated with comfort care measures.

## 4. Conclusion

Our case highlights a rare presentation of metastatic pleural Ewing sarcoma, in a patient who had previously been treated for skeletal Ewing sarcoma and was thought to be in remission. It represents an unusual presentation of this metastatic tumour with spontaneous haemothorax, and stresses on the significance of early diagnosis and management of haemothorax.

## Funding

No funding was received for this manuscript.

## Consent

No written consent was undertaken from the patient as the patient unfortunately passed away; furthermore, no patient identifiable information was included.

## Conflicts of Interest

The authors declare no conflicts of interest.

## Data Availability

The data that support the findings of this study are available from the corresponding author upon reasonable request.
